# Composites of Pea Protein Nanofibril and Epigallocatechin Gallate: Formation Mechanism, Structural Characterization, and Antioxidant Activity

**DOI:** 10.3390/foods14142418

**Published:** 2025-07-09

**Authors:** Hailing Zhang, Yangxuan Yang, Yuting Fan, Jiang Yi

**Affiliations:** 1 Shenzhen Key Laboratory of Food Macromolecules Science and Processing, College of Chemistry and Environmental Engineering, Shenzhen University, Shenzhen 518060, China; a17807060098@163.com (H.Z.); 18344122204@163.com (Y.Y.); 2 School of Public Health, Shenzhen University Medical School, Shenzhen University, Shenzhen 518060, China

**Keywords:** polyphenol, amyloid-like fibril, characteristics, complex, interaction

## Abstract

The EGCG/PPN composite, prepared by combining pea protein nanofibrils (PPNs) with epigallocatechin gallate (EGCG), could be used as a multifunctional nanocarrier. Compared to pea protein isolate (PPI), EGCG/PPN composites exhibited remarkably higher turbidity and zeta potential, along with similar UV spectra. Intrinsic fluorescence spectroscopy, ThT fluorescence spectroscopy, and surface hydrophobicity analysis suggested that the interactions between EGCG and PPN were primarily driven by hydrophobic forces. UV spectra indicated that the microenvironment of amino acid residues in the tertiary structure of the protein changes upon complexation, and circular dichroism (CD) revealed that the incorporation of EGCG increases the β-sheet content in the protein’s secondary structure. Analyses of DPPH and ABTS radical scavenging activity, as well as reducing power, demonstrated that the synergistic effect between EGCG and PPN did not hinder the inherent antioxidant properties of EGCG but rather enhanced them significantly. Transmission electron microscopy (TEM) images showed that the addition of EGCG reconstructed the fibril morphology, thereby affecting the properties of PPNs. Overall, the composite fabricated through the interaction between PPN and EGCG shows great potential as a nanocarrier in the processing of functional foods.

## 1. Introduction

Proteins, as essential nutrients in daily diets, are experiencing rapidly growing global demand. Plant proteins are increasingly favored by consumers due to their lower saturated fat content and environmental friendliness. Pea protein, in particular, has garnered significant interest because of its low allergenicity, relatively high nutritional value, easy accessibility, sustainability, and low cost [1]. Similar to most other vegetable proteins, nevertheless, challenges in using pea protein as a versatile ingredient in various food systems occur in terms of limitations in functionalities (such as poor solubility and emulsifying properties) due to its poor water solubility, which is primarily because of the rigid structures of subunits (i.e., legumin) [1]. Overcoming this functional limitation would lead to wider application of plant proteins in the food system. Therefore, various methods are applied to modify pea protein. Traditional functional processing of plant proteins includes heat treatment, glycosylation, fermentation, and enzymatic catalysis, which can improve protein solubility, foaming, emulsifying properties, and nutraceutical-binding and delivering capacity [2].

Amyloid-like fibrils are linear aggregates formed by the self-assembly of proteins or peptides under certain conditions (like an acidic condition with heating). They are characterized by a structural conformation consisting of a cross-linked network of β-sheets aligned parallel to the fibril axis. Nowadays, food protein-derived nanofibrils have gained various wide interest because of their high aspect ratio, robust mechanical characteristics, and numerous alterable functional groups [3,4,5]. Plant proteins can self-assemble into amyloid-like fibrils with thermal treatment under acidic conditions, which would markedly enhance their solubility, antioxidant activity, emulsifying, and gelling properties [6]. Pea protein nanofibrils (PPNs) fabricated from their native form can effectively improve various physicochemical properties of the native protein components [7].

In novel processing approaches, interactions with polyphenols can enhance the hydrophilicity and solubility of proteins, and facilitate the transformation of secondary and tertiary structures. This approach represents an effective new processing strategy, offering a promising avenue for further research and development [8]. Polyphenols represent a class of secondary metabolites that are widely distributed in a variety of plant-based foods, including vegetables, fruits, tea, and coffee. Polyphenolic compounds found in fruits and vegetables are typically classified as flavonoids and represent a significant source of dietary antioxidants for humans. These compounds play a crucial role in the prevention of various diseases [9]. In recent years, there has been a growing interest in the health-promoting benefits of polyphenolic compounds. Among these, epigallocatechin gallate (EGCG) is the most abundant and biologically active flavanol-3-ol polyphenol found in green tea [10], and EGCG exhibits excellent antioxidant capabilities and the ability to reshape the microstructure of mature protein nanofibrils [11]. Nevertheless, EGCG is prone to oxidation, degradation, or aggregation when exposed to certain pH, heating, and oxygen, which strictly restrains its commercial applications in the food system. Encapsulation is generally recognized as an effective approach to improve the stability of EGCG under harsh conditions. Among all recently reported carriers, the amyloid-like protein fibril-based nano-delivery system, due to high aspect ratio, exhibits great potential to stabilize EGCG effectively [12].

The interaction between polyphenolic compounds and amyloid-like protein fibrils has also sparked significant interest. Some scholars suggest that polyphenols can effectively bind with food proteins, forming protein–polyphenol complexes that enhance their stability [13]. EGCG can interact strongly with pea protein fibrils through hydrophobic interactions mediated by ultrasound treatment [14]. Polyphenols can reshape or cut mature amyloid-like protein fibrils into water gels at low soy protein concentrations [11]. Studies have shown that polyphenols are effectively adsorbed and deposited on the surface of amyloid-like protein fibrils, forming supramolecular nanofibrils. These nanofibrils self-assemble at multiple length scales and crosslink through non-electrostatic physical interactions, thereby altering their physicochemical properties [15]. There are numerous studies investigating the interaction between pea protein and EGCG, and the results demonstrate the great potential for pea protein–EGCG composite as a food ingredient for application in food systems. Nevertheless, research characterizing the interaction between EGCG and PPN is currently limited.

This study aims to investigate the impact of incorporating EGCG on PPN through composite formation, seeking to understand how EGCG influences the structural and physicochemical properties of PPNs, and the interaction mechanisms between EGCG and PPN. Utilizing PPN to create various functional materials could provide sustainable and cost-effective nanocarriers for bioactive substances.

## 2. Materials and Methods

### 2.1. Materials

Pea protein isolate (PPI, ≥82% protein) was provided by Roquette (Lestrem, France). 1-Anilino-8-naphthalene-sulphonate (ANS) was purchased from Sigma-Aldrich (St. Louis, MO, USA). 2,2-Diphenyl-1-picrylhydrazyl (DPPH); 2, 2′-azino-bis (3-ethylbenzothiazoline-6-sulfonic acid) (ABTS); thioflavin T (ThT); and EGCG were purchased from Macklin Biochemical Technology (Shanghai, China). All chemicals used were analytical grade. Deionized water was applied in all assays.

### 2.2. PPI Nanofibril Fabrication

Pea protein nanofibrils (PPNs) were gained through heating (85 °C) at an acidic condition (pH 2.0). PPI powder was completely dispersed in deionized water at pH 2.0 through magnetic stirring for 2 h to ensure the full hydration of soluble protein molecules, and then subjected to centrifugation (4000× *g*, 10 min, 10 °C) to remove insoluble materials. The attained supernatant was then freeze-dried. Soluble PPI powder was redissolved in deionized water (pH 2.0) and the protein concentration was adjusted to 30 mg/mL. Thermal treatment was performed for 24 h to cause the formation of PPN in a water bath (85 °C). After that, PPN solution was immediately cooled to room temperature with an ice-water bath and stored in a refrigerator at 4 °C for further use.

### 2.3. Sodium Dodecyl Sulfate Polyacrylamide Gel Electrophoresis (SDS-PAGE)

Molecular weight distribution of PPN was measured using SDS-PAGE under a reducing condition. PPN solution was diluted to roughly 2.0 mg/mL with the loading buffer (4-fold) in the presence of (dithiothreitol (DTT)), and heated for 5 min in boiling water before loading (10 μL). The gel produced in our laboratory mainly consisted of 15% separating gel and 5% stacking gel. The protein separation was performed at 120 V. After running, the gel was stained, bleached, and photographed. PPI was used as the control.

### 2.4. Composites of PPN and EGCG

EGCG stock solution was first prepared by fully dissolving in deionized water (pH 2.0), and then added dropwise to PPN solution with various volumes to reach different [EGCG]/[PPN] mass ratios (0, 0.025, 0.05, 0.075, 0.1, 0.15, 0.2, and 0.25). Protein concentration was maintained at 1% (*w*/*v*). After addition, magnetic stirring was continued for 1 h to achieve the full complexation between PPN and EGCG [5]. All samples were then stored at 4 °C or freeze-dried for further analysis. Soluble PPI powder was used as the control.

### 2.5. Turbidity

Turbidity measurements of various PPN-EGCG composite dispersions ([EGCG]/[PPN] with mass ratios (0, 0.025, 0.05, 0.075, 0.1, 0.15, 0.2, and 0.25)) were conducted using a UV–visible spectrophotometer (UV-2600, Shimadzu, Kyoto, Japan) at 600 nm. Final protein concentration was diluted to 1.0 mg/mL before determination. Soluble PPI powder was used as the control.

### 2.6. Zeta Potential

The ζ-potential of PPN-EGCG composites at various [EGCG]/[PPN] mass ratios (0, 0.025, 0.05, 0.075, 0.1, 0.15, 0.2, and 0.25) was measured using a Zetasizer (Malvern Company, Worcestershire, UK). Prior to measurement, samples were diluted to a final protein concentration of 1 mg/mL in deionized water (pH 2.0). The refractive indexes were set at 1.450 and 1.330 for composites and deionized water, respectively. Measurements were conducted with an equilibration time of 120 s at 25 °C. Soluble PPI powder was used as the control.

### 2.7. UV Spectroscopy

UV spectra of the samples were scanned using a UV-2600 spectrophotometer in the wavelength range of 250–350 nm. The [EGCG]/[PPN] mass ratios tested were 0.025, 0.05, 0.075, 0.1, 0.15, 0.2, and 0.25, with a protein concentration of 1 mg/mL. Deionized water at pH 2 served as the blank. Soluble PPI powder was used as the control.

### 2.8. Fluorescence Spectroscopy

PPN-EGCG composite dispersions with varied [EGCG]/[PPN] mass ratios (0, 0.025, 0.05, 0.075, 0.1, 0.15, 0.2, and 0.25) were diluted to a protein concentration of 1 mg/mL in deionized water (pH 2.0). Fluorescence spectra were recorded using a fluorescence spectrophotometer (F-7000, Hitachi, Tokyo, Japan) with an excitation wavelength of 280 nm (slit width: 5.0 nm) and an emission wavelength range of 300 to 500 nm (slit width: 5.0 nm).

### 2.9. Circular Dichroism (CD)

CD spectra of PPN-EGCG composites at different [EGCG]/[PPN] mass ratios (0, 0.025, 0.05, 0.075, 0.1, 0.15, 0.2, and 0.25) were recorded using a CD spectropolarimeter (J-815, Jasco, Hachioji, Japan) in a 2 mm quartz cuvette through the wavelength range of 190–260 nm. The scan speed was set at 100 nm/min with a bandwidth of 1 nm. The measurements were performed at 25 °C with 5 repetitions. Baseline data attained with pH 2.0 HCl solution were subtracted from each spectrum. The protein concentration was maintained at 0.2 mg/mL.

### 2.10. Thioflavin T (ThT) Assay

The thioflavin T (ThT) assay was carried out following the method described by Nilsson, with minor modifications [16]. ThT stock solution (0.8 mg/mL) was gained by dissolving in phosphate buffer (PB, 10 mM, pH 7.0) containing 150 mM NaCl. ThT working solution was attained through 50-fold dilution of this stock solution. All samples were diluted in deionized water (pH 2.0). Subsequently, 3 mL of the ThT working solution was mixed with 30 μL of each sample and incubated for 3 min. Fluorescence intensity was measured using a fluorescence spectrophotometer at an excitation wavelength of 390 nm in the emission wavelength range of 410–550 nm.

### 2.11. Surface Hydrophobicity

Hydrophobicity (H_0_) of PPN-EGCG composites as a function of [EGCG]/[PPN] mass ratios (0, 0.025, 0.05, 0.075, 0.1, 0.15, 0.2, and 0.25) was examined based on the detailed approach previously using ANS as a fluorescent probe method, with slight modifications [17,18]. PPN-EGCG composites with different protein concentrations ranging from 0.01 to 0.2 mg/mL were achieved by dilution with deionized water (pH 2.0). Subsequently, 20 μL of ANS reagent was supplemented to each diluted solution (2 mL) and incubated at room temperature for 15 min. Fluorescence intensity was examined using a fluorescence spectrophotometer (F7000, Hitachi, Japan) with an excitation wavelength set at 390 nm (slit width = 5 nm) and an emission wavelength of 470 nm (slit width = 5 nm). H_0_ was determined by calculating the initial slope of the fluorescence intensity plotted against the corresponding protein concentration.

### 2.12. Transmission Electron Microscopy (TEM)

EGCG/PPN composite dispersions with varied [EGCG]/[PPN] mass ratios (0, 0.025, 0.05, 0.075, 0.1, 0.15, 0.2, and 0.25) were diluted 500-fold with deionized water (pH 2). Each diluted sample was then incubated on a copper grid for 120 min and air-dried at room temperature for one day. The microscopic morphology of each sample was recorded using a transmission electron microscope (JEM-2100, JEOL Co., Ltd., Tokyo, Japan) at an accelerating voltage of 100 kV.

### 2.13. Antioxidant Activity

#### 2.13.1. DPPH Scavenging Activity

DPPH free radical scavenging assay has been used extensively to evaluate the antioxidant capacity of food ingredients. PPN-EGCG composites with various [EGCG]/[PPN] mass ratios (0, 0.025, 0.05, 0.075, 0.1, 0.15, 0.2, and 0.25, *w*/*w*) at a constant protein concentration of 0.2 mg/mL were used for DPPH scavenging activity evaluation, corresponding to EGCG concentrations of 0, 5, 10, 15, 20, 30, 40, and 50 μg/mL. Similarly, a series of EGCG aqueous solutions (pH 2) with the same concentrations of EGCG in PPN-EGCG composites were also prepared for DPPH scavenging activity determination. The reaction mixture (total volume 2 mL) consisted of 150 μL of each sample and 1.85 mL of 0.1 mM DPPH ethanol solution. After thorough mixing, the mixture was allowed to stand in the dark at room temperature for 30 min. Absorbance (As) was measured at 517 nm using a UV-2600 spectrophotometer. The absorbance of a mixture of 150 μL water and 1.85 mL DPPH solution at 517 nm (Ac) was also measured. The DPPH radical scavenging percentage was calculated using the following formula:

(1) DPPH radical scavenging percentage (%) = (Ac − As)/Ac × 100%

#### 2.13.2. ABTS Radical Scavenging Activity

ABTS radical scavenging activity of EGCG/PPN composites at different mass ratios ([EGCG]/[PPN] = 0, 0.025, 0.05, 0.075, 0.1, 0.15, 0.2, and 0.25, *w*/*w*) was examined using the method previously described. ABTS solution (7 mM) was prepared by dispersing in deionized water, and potassium persulfate solution (4.9 mM) was attained by dissolving in deionized water. Equal volumes of these solutions were mixed thoroughly, and the mixture was kept in the dark at room temperature (25 °C) for 12 h to generate ABTS^+^ radicals (resulting in a dark green color). The produced ABTS^+^ radical solution was diluted with PBS solution to an absorbance of 0.700 ± 0.002 at λ = 734 nm (designated as Ac), and experiments were conducted using this diluted ABTS^+^ radical solution. Then, 100 μL of each sample (0.2 mg/mL protein concentration) was mixed with 1.9 mL of ABTS^+^ radical solution, followed by incubation in the dark at room temperature for 30 min. Subsequently, absorbance (As) of the reaction mixture was examined at 734 nm using a UV–visible spectrophotometer. ABTS radical scavenging activity (%) was calculated using the following formula:

(2) ABTS radical scavenging activity (%) = (Ac − As)/Ac × 100%

#### 2.13.3. Reducing Power

Reducing power of EGCG/PPN composites at different [EGCG]/[PPN] mass ratios (0, 0.025, 0.05, 0.075, 0.1, 0.15, 0.2, and 0.25) was tested. Generally, 0.5 mL of sample (protein concentration 0.5 mg/mL) was mixed with 1.25 mL of 0.2 M PB (pH 6.6) and 1.25 mL of potassium ferricyanide (10 g/L) and kept at 50 °C for 20 min. Subsequently, 1.25 mL of 10% (*w*/*v*) trichloroacetic acid (TCA) was added and completely mixed, followed by centrifugation at 1500× *g* for 10 min. A 1.25 mL aliquot of the supernatant was then mixed with 0.25 mL of FeCl_3_. Finally, after sitting for 10 min at room temperature, the absorbance at 700 nm was then measured using a UV–visible spectrophotometer.

### 2.14. Statistical Analysis

Statistical significance (*p* < 0.05) was determined using SPSS Statistics (SPSS 19.0, Chicago, IL, USA) to perform one-way analysis of variance (ANOVA) to compare the means.

## 3. Results and Discussion

### 3.1. SDS-PAGE

**Figure 1:**
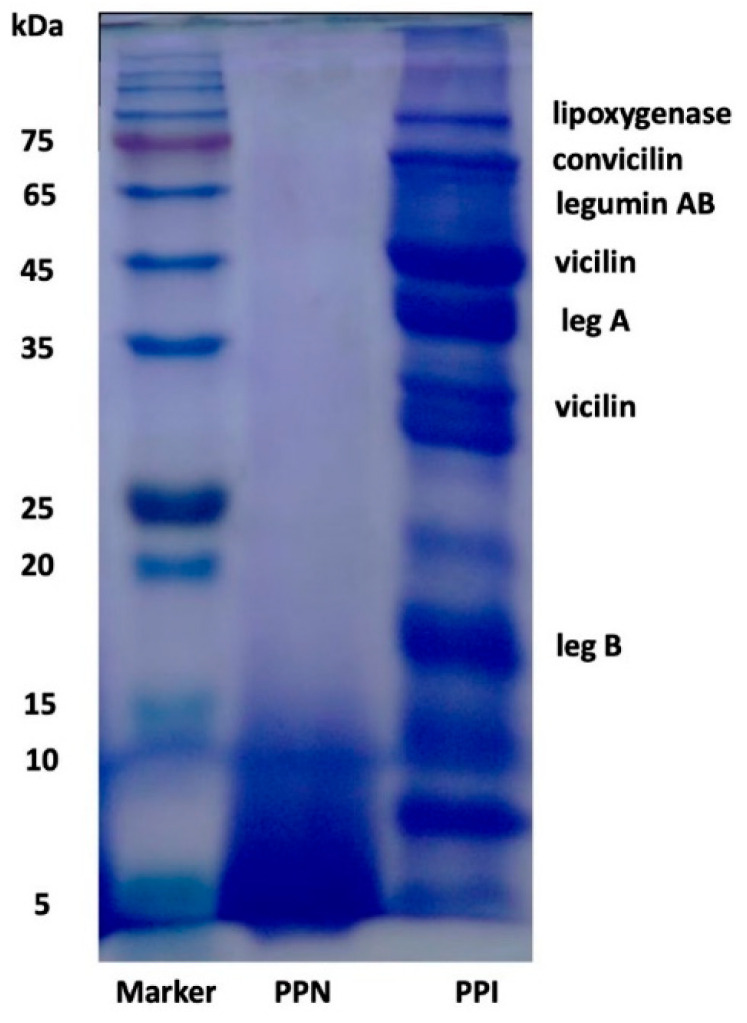
SDS-PAGE image of pea protein isolate (PPI) and PPI nanofibrils (PPNs) exposed to 24 h heating at 25 °C (pH 2.0).

It has been demonstrated that amyloid-like nanofibril formation of food proteins, especially globular proteins, is initiated by consecutive hydrolysis of proteins into polypeptides induced by thermal treatment at pH 2.0, followed by self-assembly of some released peptides into nanofibrils. SDS-PAGE was performed to probe the Mw alteration of pea protein under heating in an acidic condition. The dominant bands of pea protein were convicilin (about 70 kDa), legumin AB (60 kDa), legumin (approximately 40, and 20 kDa), and vicilin (roughly 50, and 35–20 kDa) under a reducing condition (Figure 1), consistent with the previous report [7]. Compared with other subunits, the amount of vicilin was the highest. The band located at about 100 kDa could be lipoxygenase [19]. Minor protein aggregates (>150 kDa) accumulated on the top of the gel can be observed, probably because of the existence of subunit aggregates produced during the commercial extraction process. This demonstrates the main fractions of pea proteins (legumin, vicilin, and convicilin) were still present before thermal treatment. Nevertheless, all major bands, including lipoxygenase and protein aggregates, disappeared with 24 h heating at pH 2.0, confirming the occurrence of pea protein hydrolysis, a prerequisite for the formation of PPN. A broad band with the Mw between 5 and 10 kDa was formed, corresponding to released peptides and formed fibrils derived from pea protein, in line with previous study [7,20]. The hydrolysis of proteins and released peptides during heating under the acidic condition could dominate the fibrillation process.

### 3.2. Turbidity

**Figure 2:**
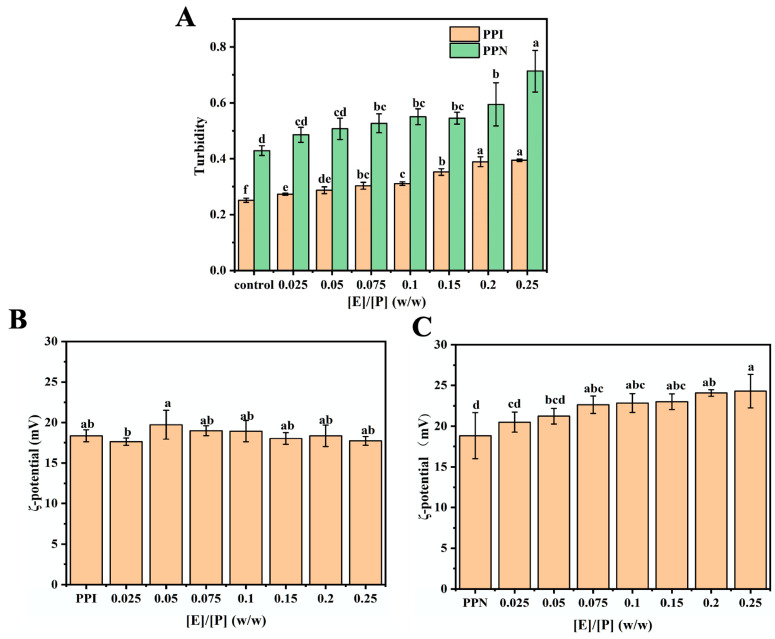
Turbidity (**A**) of EGCG/PPI and EGCG/PPN at different mass ratios ([E]/[P] = 0, 0.025, 0.05, 0.075, 0.1, 0.15, 0.2, and 0.25, *w*/*w*); zeta potential of EGCG/PPI (**B**) and EGCG/PPN (**C**) at different mass ratios ([E]/[P] = 0, 0.025, 0.05, 0.075, 0.1, 0.15, 0.2, and 0.25, *w*/*w*). Various letters (a, b, c, d, e, and f) denote significant differences at a level of *p* < 0.05 in each bar chart with the same color columns.

The aggregation of proteins and polyphenols can be assessed through turbidity measurements, as particle size and concentration are related to the degree of light scattering [21]. At the same protein concentration, the turbidity of PPN alone at pH 2 was pronouncedly higher than that of PPI (native) (Figure 2A). This enhancement is probably due to the higher degree of nanofibril aggregation, which is formed by the self-assembly of polypeptides resulting from the acid–heat hydrolysis of pea protein. In addition, pH can pronouncedly influence protein charge and alter their interactions, playing a crucial role in the turbidity of protein complexes [22]. The turbidity of both PPN-EGCG and PPI-EGCG composites markedly rose, increasing the EGCG content. Moreover, the turbidity of PPN-EGCG composites was much higher than that of PPI-EGCG complexes. This manifests that polyphenols can promote protein aggregation, and their higher combination with protein nanofibrils could contribute to a higher degree of aggregation and the formation of larger complex particles, as evidenced by the TEM graphs shown below.

### 3.3. Zeta Potential

Zeta potential is a valuable tool for elucidating the surface charge density and interfacial properties of proteins. The incorporation of EGCG has a negligible impact on the zeta potential of PPI-EGCG composites (Figure 2B). This effect is likely attributed to the lack of charge on EGCG molecules and the predominant non-electrostatic nature of the interaction between EGCG and PPI. The pKa values of EGCG are around 7.65 for the first hydroxyl group deprotonation. In this study, zeta potential of all the samples was measured at pH 2.0, much lower than the pKa of EGCG, suggesting it is electrically neutral (a lack of charge). Similarly, there is also minimal alteration in the surface charge of PPI following polyphenol binding [21]. Protein molecules undergo hydrolysis when exposed to acid and heat treatment, resulting in the formation of polypeptides and subsequent self-assembly into nanofibrils. This process would lead to the exposure of charged amino groups on the protein surface. These positively charged groups participate in the assembly of protein nanofibrils, thereby increasing the surface charge of PPN and resulting in a higher zeta potential value (Figure 2C). In addition, the rise of zeta potential is mostly likely due to the exposure of more charged amino acid residues induced by hydrophobic interactions between the aromatic rings of polyphenols (chlorogenic acid) and the active sites of proteins (soy protein fibrils) [23]. Furthermore, the incorporation of EGCG also contributes to a significant increase in the surface charge of bovine serum albumin nanofibrils [24]. The absolute zeta potential values of PPN with EGCG were higher than 20 mV, indicating these fibrils exhibited enhanced electrostatic repulsion and steric hindrance [25]. Strong electrostatic repulsion could help maintain the stability of EGCG/PPN composites during storage or application.

### 3.4. UV Spectroscopy

**Figure 3:**
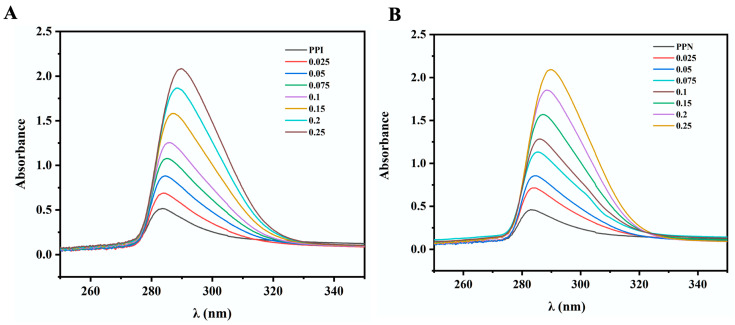
UV spectra of EGCG/PPI (**A**) and EGCG/PPN (**B**) at different mass ratios ([E]/[P] = 0, 0.025, 0.05, 0.075, 0.1, 0.15, 0.2, and 0.25, *w*/*w*).

Changes in the microenvironment of hydrophobic amino acid residues in proteins can be revealed through variations in the intensity and position of UV absorption peaks [26]. As shown in Figure 3A, the intensity of the absorption peaks increased with the rising concentration of EGCG, implying that the degree of complexation between EGCG and PPI or PPN molecules was expanding. The occurrence of a red shift of UV absorption peaks suggested that the changes in the microenvironment of hydrophobic amino acid residues in PPN upon interaction with polyphenols were consistent with the alterations observed in PPI. The UV spectra of both EGCG/PPI and EGCG/PPN composites are also consistent with previous reports [21]. Interestingly, the UV spectra of pea protein were almost identical to those of PPN (Figure 3B), with similar maximum absorption wavelengths, probably reflected that the mode of complexation between EGCG and PPI, as well as PPN, was similar.

### 3.5. Fluorescence Spectroscopy

**Figure 4:**
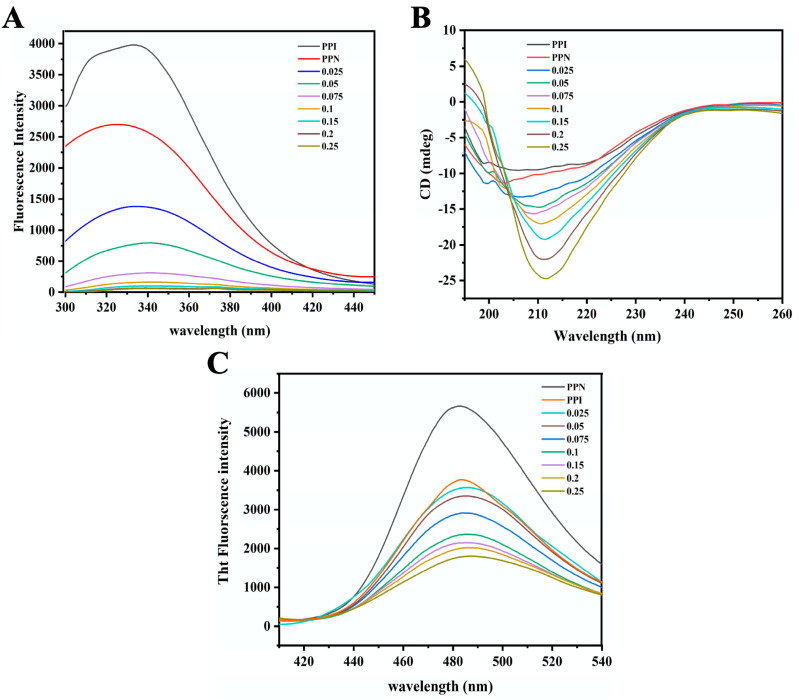
Fluorescence spectra (**A**), CD (**B**), and Thioflavin T (ThT) fluorescence spectra (**C**) of PPI, PPN, and EGCG/PPN composites at different mass ratios ([E]/[P] = 0.025, 0.05, 0.075, 0.1, 0.15, 0.2, and 0.25, *w*/*w*).

Monitoring changes in the tertiary structure of proteins using fluorescence spectroscopy at an excitation wavelength of 280 nm is a classic method, primarily due to the polarity alteration in the microenvironment of tryptophan (Trp) and tyrosine (Tyr) residues. Aromatic amino acids, such as Trp and Tyr, are typically found in the hydrophobic regions of PPI. PPI exhibits characteristic peaks in the fluorescence spectrum (around 335 nm) (Figure 4A). The maximum fluorescence intensity of PPN was significantly lower than that of PPI, evidently demonstrating the formation of protein fibril aggregates, which partially blocked the fluorescence intensity induced by aromatic amino acid residues, consistent with the recent result that a significant decrease in the fluorescence intensity of bovine serum albumin nanofibrils under heating in an acidic condition has also been observed [24]. The decline in fluorescence intensity is probably due to the exposure of Trp/Tyr to polar solvents or the burying of the dominant role of aromatic residues in the self-assembly of fibrils [14].

Upon the addition of EGCG, the fluorescence intensity of PPN dramatically dropped, primarily due to the interaction between EGCG and PPN. As the amount of EGCG increased, the fluorescence intensity progressively became lower, suggesting that the binding of EGCG to PPNs may increase the polarity of the local microenvironment or interact with Trp/Tyr, leading to fluorescence quenching. Polyphenolic compounds can enhance the polarity of the microenvironment, thereby reducing the fluorescence intensity of proteins [27]. The addition of EGCG caused a pronounced red shift in the λ_max_, implying an increase in the polarity of the microenvironment around Trp/Tyr in EGCG/PPN, with aromatic amino acid residues exposed to a more hydrophilic environment [28].

The binding constant Ks and binding number n for the static quenching of PPNs and PPI with various concentrations of EGCG can be calculated according to the following formula:

(3) $$log\left[\frac{F_{0}-F}{F}\right]=logK_{s}+nlog\left[EGCG\right]$$

where F_0_ and F represent the fluorescence intensities (λ_max_) with or without EGCG; [EGCG] refers to the EGCG concentration, and Ks represents a constant, equivalent to the reciprocal of the quencher concentration as the fluorescence emission intensity (λ_max_) decreases to 50%. The binding constant of PPN for EGCG was calculated to be 5.24 × 10^9^ M^−1^, remarkably higher than the binding constant of PPI with EGCG (1.08 × 10^8^ M^−1^), suggesting fibrillation would considerably enhance the binding capacity of PPN with EGCG due to exposed hydrophobic groups and specific structure characteristics. The corresponding number of binding sites of PPN for EGCG (n) was calculated to be 2.27, pronouncedly higher than that of PPI (1.95).

### 3.6. CD

The alterations in the secondary structure content of PPN-EGCG composites as a function of EGCG/PPN mass ratio were investigated using a CD spectrometer. Two typical negative peaks around 220 and 208 nm were observed for PPI (Figure 4B), an indicator of a protein rich in α-helical structures. The secondary structure compositions calculated using the CONTINLL analysis method are depicted in Table 1, and PPI mainly consisted of 56% α-helix, 18% β-sheet, 9% β-turn, and 18% random coil. Whereas, PPN was primarily composed of 44% α-helix, 24% β-sheet, 7% β-turn, and 14% random coil. The exposure of buried residues (hydrophobic amino acids) occurred during acid–heat- induced protein unfolding and hydrolysis, leading to the formation of PPNs with raised β-sheet structures. Upon addition of EGCG, the peak of PPN-EGCG composites shifted from 204 nm to approximately 211 nm, demonstrating the changes in PPNs’ secondary structures. The increased maximum negative mean residue ellipticity indicated the formation of more β-sheet structures. The incorporation of EGCG led to the pronounced decline in α-helix, and β-turn content, but the remarkable rise in β-sheet content, demonstrating that some of the α-helix structure might have been altered into β-sheet structure, in line with a recent report [21]. The addition of EGCG to trypsin also contributes to remarkable rise in the negative molar ellipticity of trypsin [29]. This result suggests that EGCG would influence the secondary structures of PPNs, mainly due to the binding of EGCG to the hydrophobic amino acid regions in the α-helix structure, leading to the alterations in the spatial conformation of PPN molecules. There were no marked impacts on random coil content. As the mass ratio [E]/[P] increases, the interaction between EGCG and PPN causes the α-helical content of PPN to continue decreasing, while the β-sheet content further increases. When the [E]/[P] mass ratio reaches or exceeds 0.075, the β-sheet content exceeds 30%. The β-sheet content would remarkably impact the functionality of food proteins. For example, a higher β-sheet content could result in the enhancement of thermal stability and relative low digestibility [30,31]. The outcomes demonstrate that the interaction between EGCG and PPN alters the protein’s secondary structure, increasing the content of β-sheets.

### 3.7. ThT Fluorescence

Thioflavin T (ThT) is a specific dye utilized to assess the conventional structural characteristics (β-sheet) of protein fibrils [16]. ThT primarily enhances fluorescence intensity through hydrophobic interactions with highly ordered β-structures of protein-derived fibrils. As illustrated in Figure 4C, the initial fluorescence intensity of PPI was relatively low. However, there was a notable increase in ThT fluorescence intensity in PPN following thermal treatment in an acidic condition, indicating the formation of fibrillar aggregates. This observation is consistent with previous studies [32]. Similarly, acid–heat treatment would result in the formation of lentil protein fibrils, accompanied by a notable increase in ThT fluorescence intensity [33]. Upon the addition of EGCG, a notable decrease in fluorescence intensity was observed, suggesting that the hydrophobic interaction between EGCG and PPN may have competitively bound the binding sites between protein fibrils and ThT, thereby lowering ThT fluorescence intensity [34]. In addition, EGCG may interact with PPN, regulating the conformation of PPNs and therefore decreasing the fluorescence intensity [35]. Furthermore, EGCG might cause partial depolymerization or disaggregation of the fibrils, thereby contributing to the observed decrease in ThT fluorescence. Finally, the alterations in the PPN surface charges could remarkably impact the fluorescence intensity. The affinity between negative surface charges of PPNs and the ThT molecules would lead to the occurrence of a remarkable negative relationship between the surface zeta potential of PPNs and ThT fluorescence intensity.

The rise in EGCG concentration led to the gradual decline in ThT fluorescence intensity of PPN, emphasizing that the impact of EGCG on PPN was concentration-dependent. Notably, the catechol unit of EGCG is prone to self-oxidation, owing to the structure–activity relationship between caffeoyl–quinic acid derivatives, which would lead to the formation of o-quinone. The formation of a covalent bond between o-quinone and certain residues of PPN would disrupt the stability of the β-sheets, and resulting in reducing ThT fluorescence intensity.

### 3.8. Surface Hydrophobicity (H_0_)

**Figure 5:**
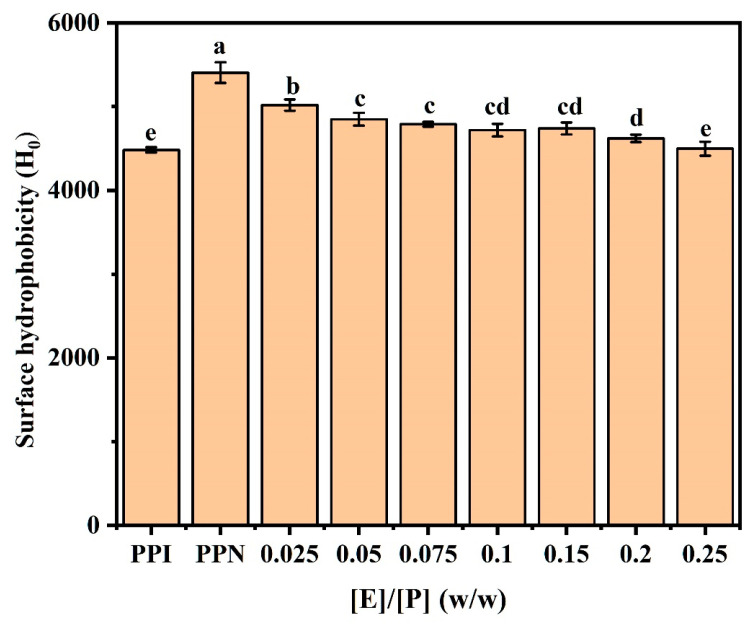
Surface hydrophobicity (H_0_) of PPI, PPN, and EGCG/PPN complexes at different mass ratios ([E]/[P] = 0.025, 0.05, 0.075, 0.1, 0.15, 0.2, and 0.25, *w*/*w*). Various letters (a, b, c, d, and e) denote significant differences at a level of *p* < 0.05.

As a fluorescence probe, ANS preferentially binds to hydrophobic sites of food proteins and is broadly applied to assess the quantity and distribution of hydrophobic patches on sample surfaces [36]. Surface hydrophobicity reflects non-polar hydrophobic groups on protein molecule surfaces and can remarkably influence the functionalities such as emulsifying properties and gelling characteristics [37]. PPN formation enhanced the surface hydrophobicity of pea protein (Figure 5), due to the release of peptide and the exposure of hydrophobic groups during hydrolysis and self-assembly caused by heating in an acidic condition, consistent with our previous study [7]. The addition of EGCG resulted in the progressive decline in PPN surface hydrophobicity, implying a reduction in hydrophobic groups. At low concentrations, EGCG would compete with ANS for hydrophobic sites, while excess EGCG and its oxidative or aggregated products at relatively high concentrations may induce protein unfolding, leading to protein aggregation and shielding of internal hydrophobic groups in PPN [38]. This result demonstrates that the binding of EGCG to PPN is primarily through hydrophobic interactions. Some studies suggest that EGCG, with its aromatic rings and numerous hydroxyl groups, interacts hydrophobically with protein molecules [39]. Recent research also illustrates that the main driving forces for the complexation between soy protein fibrils and chlorogenic acid are hydrogen bonding or hydrophobic interactions [23].

### 3.9. TEM

**Figure 6:**
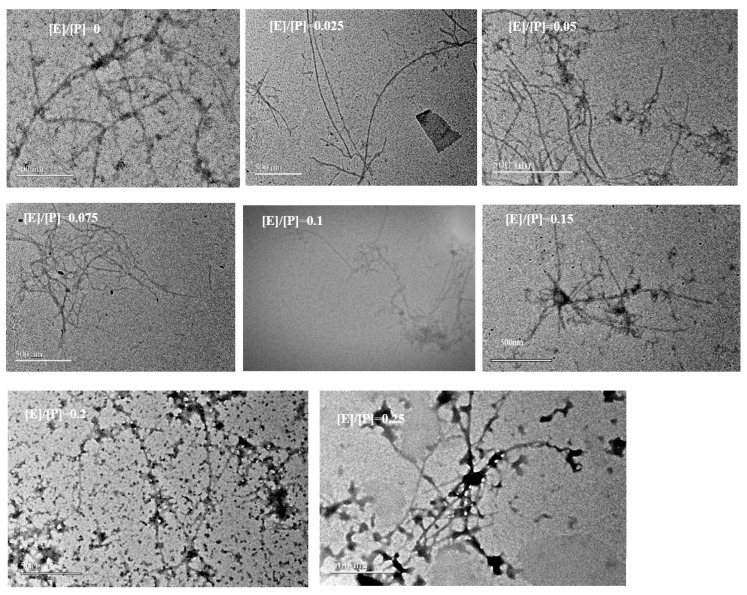
TEM of PPNs and EGCG/PPN composites at various mass ratios ([E]/[P] = 0.025, 0.05, 0.075, 0.1, 0.15, 0.2, and 0.25, *w*/*w*). Scale bar represents 500 nm.

A transmission electron microscope was employed to observe the microstructure and morphology of PPN-EGCG composites at different [EGCG]/[PPN] mass ratios (Figure 6). Pea protein, heated under acidic conditions for 24 h, could form mature nanofibrils. PPNs were semiflexible, linear, but curly, exhibiting a worm-like appearance, which was comparable to that of the nanofibrils fabricated with soy protein at pH 2.0 under heating [40], but varying from nanofibrils produced with α-lactalbumin [41]. The length of the PPNs varied in the range of about 1–4 μm, with an estimated thickness of approximately between 4 nm and 10 nm, in agreement with other protein-derived nanofibrils such as whey protein isolate [42], lysozyme [11], and β-lactoglobulin [43], which possess thicknesses between 5 nm and 10 nm.

Addition of low concentrations of EGCG caused some morphological changes in PPNs, resulting in finer and elongated nanofibrils. Previous studies have reported similar structural alterations in lysozyme fibrils upon EGCG addition [11]. As the [EGCG]/[PPN] mass ratio increased gradually, EGCG could induce the crosslinking of PPNs to form larger and more stable network structures. Concurrently, the elongated fibril morphology began to exhibit curvature, and PPNs showed increased aggregation and intertwining, indicating a concentration-dependent effect of EGCG on protein fibrils morphology. Earlier research has documented EGCG’s capability to reshape soy protein fibril structures [44].

### 3.10. Antioxidant Activity

**Figure 7:**
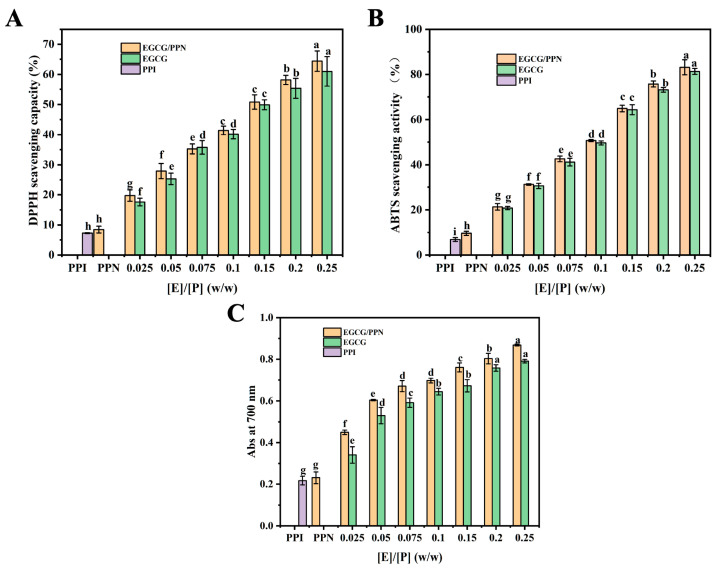
DPPH scavenging capacity (**A**), ABTS scavenging activity (**B**), and reducing power (**C**) of EGCG and EGCG/PPN composites at various mass ratios ([E]/[P] = 0.025, 0.05, 0.075, 0.1, 0.15, 0.2, and 0.25, *w*/*w*). PPI and PPN (10 mg/mL) were used as controls. Various letters (a, b, c, d, e, f, g, and h) denote significant differences at a level of *p* < 0.05 in each bar chart.

The important role of active hydroxyl groups of polyphenols in antioxidant activity can significantly enhance the antioxidant capability of proteins through interactions between polyphenolic compounds and proteins [45]. The antioxidant properties of PPI, PPN, EGCG, and PPN-EGCG composites were investigated using DPPH, ABTS, and reducing power assays. The DPPH scavenging abilities of various composites are shown in Figure 7A.

DPPH scavenging ability of PPI was relatively weak. The value at 0.2 mg/mL was negligible (0.5%). At 10 mg/mL concentration, the value was only 7.2%. However, PPN exhibited higher DPPH scavenging activity compared to PPI, possibly due to the exposed amino acid groups within the pea protein isolate fibers and their high aspect ratio network structure, facilitating interactions between amino acid residues and radicals [7]. The DPPH scavenging ability of PPN-EGCG composites was 42.5% ([E]/[P] = 0.1, *w*/*w*), whereas the value for EGCG alone (crystal) was 38.1%, indicating that the DPPH scavenging ability of PPN-EGCG composites was much stronger than that of EGCG alone (crystal) at the same concentration, likely due to interactions between EGCG and PPN, with the latter’s high surface area at the nanoscale, which could promote the reaction between EGCG and DPPH. This result may suggest the synergistic antioxidant capacity between PPN and EGCG. EGCG could reactivate the active amino acid residues of PPN through hydrogen atom transfer, which is vital mechanism for dietary antioxidants. A recent report demonstrates that whey protein fibrils could considerably ameliorate the antioxidant properties of curcumin [46], and Bu et al. shows that curcumin encapsulated with hawthorn pectin and Tenebrio Molitor protein composite hydrogel notably enhances antioxidant capabilities [47]. Nevertheless, no remarkable variations were observed between EGCG and PPN-EGCG composites at high EGCG concentrations ([E]/[P] = 0.15, 0.20, and 0.25, *w*/*w*).

Similar results were observed in ABTS^+^ radical scavenging and reducing power assays (Figure 7B,C), implying a significant enhancement in antioxidant activity following complexation with PPN. DPPH and ABTS radical scavenging capacities and reducing power all exhibited concentration-dependent relationships, wherein increasing concentrations enhanced protein antioxidant capabilities. These outcomes suggest that the synergistic effects between EGCG and PPN do not hinder EGCG’s inherent antioxidant performance but rather enhance it to some extent.

## 4. Conclusions

Both PPI and PPN can interact with EGCG to form composites. Compared to PPI-EGCG composites, PPN-EGCG composites exhibited significantly higher turbidity and zeta potential, but similar UV–vis spectra. The binding of PPN with EGCG is primarily driven by hydrophobic interactions. Upon complexation, the microenvironment of amino acid residues altered remarkably, increasing β-sheet content in PPN while reducing surface hydrophobicity and significantly enhancing antioxidant capacity. TEM images revealed that the addition of EGCG altered the morphology of PPNs; lower concentrations of EGCG result in finer nanofibrils. With increasing EGCG content, crosslinking between EGCG and PPNs forms larger and more stable network structures. Consequently, elongated fibrils morphologies exhibit curvature, aggregation, and intertwining, thereby influencing the properties of PPNs. Overall, PPNs show substantial potential as nanocarriers in functional food processing.

## Figures and Tables

**Table 1:** Secondary structure compositions (α-helix, β-sheet, β-turn, and random coil) of PPN at different [E]/[P] mass ratios (0, 0.025, 0.05, 0.075, 0.1, 0.15, 0.2, and 0.25). PPI was used as control.

[EGCG]/[PPN] Ratio (*w*/*w*)	Content (%)			
	α-Helix	β-Sheet	β-Turn	Random Coil
PPI	56	18	9	18
PPN	44	24	7	14
0.025	46	25	8	21
0.05	45	27	8	21
0.075	42	30	8	21
0.1	42	31	7	21
0.15	40	32	8	21
0.2	37	33	7	23
0.25	36	35	6	23

## Data Availability

The original contributions presented in the study are included in the article; further inquiries can be directed to the corresponding author.
